# Comparative Analysis of the Clarus *Aspergillus* Galactomannan Enzyme Immunoassay Prototype for the Diagnosis of Invasive Pulmonary Aspergillosis in Bronchoalveolar Lavage Fluid

**DOI:** 10.1007/s11046-024-00876-9

**Published:** 2024-07-18

**Authors:** Sarah Sedik, Johannes Boyer, Matthias Egger, Karl Dichtl, Juergen Prattes, Florian Prüller, Martin Hoenigl

**Affiliations:** 1 Division of Infectious Diseases, Department of Internal Medicine, Medical University of Graz, ECMM Excellence Center, Auenbruggerplatz 15, 8036 Graz, Austria; 2 Translational Mycology, Medical University of Graz, Graz, Austria; 3 Diagnostic and Research Institute of Hygiene, Microbiology and Environmental Medicine, Medical University of Graz, Graz, Austria; 4 Clinical Institute of Medical and Chemical Laboratory Diagnostics, Medical University of Graz, Graz, Austria; 5 BioTechMed-Graz, Graz, Austria

**Keywords:** Invasive pulmonary aspergillosis, Galactomannan, BALF, Enzyme-linked immunoassay, Diagnostics

## Abstract

**Background:**

Galactomannan (GM) testing using Platelia Aspergillus enzyme immunoassay (Platelia AGM) from bronchoalveolar lavage fluid (BALF) aids in early diagnosis of invasive pulmonary aspergillosis (IPA). Globally, only a minority of laboratories have the capability to perform on-site GM testing, necessitating accessible and affordable alternatives. Hence, we conducted a comparative evaluation of the new clarus Aspergillus GM enzyme immunoassay prototype (clarus AGM prototype) with Platelia AGM using BALF samples.

**Methods:**

This is a single-center, prospective, cross-sectional study, where Platelia AGM testing was routinely performed followed by clarus AGM prototype testing in those with true positive or true negative AGM test results according to the 2020 EORTC/MSG and the 2024 FUNDICU consensus definitions. Descriptive statistics, ROC curve analysis, and Spearman’s correlation analysis were used to evaluate analytical performance of the clarus AGM prototype assay.

**Results:**

This study enrolled 259 adult patients, of which 53 (20%) were classified as probable IPA, while 206 did not fulfill IPA-criteria. Spearman's correlation analysis revealed a strong correlation between the two assays (rho = 0.727, *p* < 0.001). The clarus AGM prototype had a sensitivity of 96% (51/53) and a specificity of 74% (153/206) for differentiating probable versus no IPA when using the manufacturer recommended cut-off. ROC curve analysis showed an AUC of 0.936 (95% CI 0.901–0.971) for the clarus AGM prototype, while the Platelia AGM yielded an AUC of 0.918 (95% CI 0.876–0.959).

**Conclusions:**

Clarus AGM prototype demonstrated a strong correlation and promising test performance, comparable to Platelia AGM, rendering it a viable alternative in patients at risk of IPA.

## Introduction

Invasive aspergillosis is a severe fungal infection caused by *Aspergillus* spp., with potential life-threatening consequences. Annually, over 2 million people develop invasive aspergillosis, with several hundreds of million individuals considered to live with risk factors for invasive pulmonary aspergillosis (IPA) [1–3]  is the most prevalent manifestation of *Aspergillus* associated diseases [2], primarily triggered by the inhalation of *Aspergillus* spores. *Aspergillus fumigatus* is by far the dominant *Aspergillus* species causing bronchopulmonary disease in humans [4, 5]. With mortality rates ranging from 30 to 60%, IPA predominantly affects immunocompromised individuals, including those with hematological or solid organ malignancies, hematopoietic stem-cell transplant recipients, solid organ transplant recipients, and those experiencing prolonged neutropenia [6, 7]. Moreover, IPA can manifest in patients with chronic obstructive pulmonary disease and individuals with substantial corticosteroid exposure [8, 9]. Notably, there is an increasing incidence of IPA in intensive care units with severe respiratory viral infections, such as influenza, and more recently, coronavirus disease 2019 [10–13].

Timely and accurate diagnosis of IPA, followed by prompt treatment initiation, is crucial for survival [14, 15]. However, early diagnosis remains challenging due to the absence of specific clinical and radiological signs, as well as immunological markers, necessitating additional mycologic diagnostics [16–18]. These include fungal culture from sterile site samples and respiratory specimens (primarily from bronchoalveolar lavage fluid [BALF]), the identification of fungal biomarkers, polymerase chain reaction (PCR)-based techniques and, ideally, histopathological examination of infected tissue. All these modalities come along with certain limitations in terms of diagnostic performance and hard turn-around-times. The most widely used biomarker for diagnosing IA is galactomannan antigen (GM-Ag), which is typically tested with the Platelia Aspergillus enzyme immunoassay (Platelia AGM) [19]. The European Organization for Research and Treatment of Cancer/Mycoses Study Group (EORTC/MSG) consensus definitions include the detection of *Aspergillus* GM-Ag from BALF samples as a criterion to establish the diagnosis of probable IPA [20]. Despite the effectiveness of GM-Ag testing in BALF, only a minority of laboratories has the option to perform on-site Platelia AGM testing [21–23]. While alternative tests, including the Euroimmun GM Enzyme Immunoassay [24] as well as point of care tests for GM-Ag or GM-like antigens like the *Aspergillus* LFA and LFD [25–27], have been shown to be reliable alternatives to the Platelia AGM, more alternatives are needed to ensure competitive pricing of the assays. This emphasizes the need for more accessible alternatives to aid in the diagnosis of IPA.

The clarus Aspergillus AGM Enzyme Immunoassay Prototype (clarus AGM prototype (Ref AGM101), IMMY, Norman, Oklahoma) is an IVD-certified sandwich enzyme-linked immunosorbent assay that has been validated for qualitative detection of *Aspergillus* GM-Ag in BALF and serum samples. In this single-center, prospective, cross-sectional study, the primary objective was to evaluate and compare the analytic performance of the novel clarus AGM prototype assay in comparison to the established Platelia AGM (Bio-Rad, Marnes-la-Cocquette, France) assay.

## Material and Methods

A total of 259 BALF samples were collected prospectively from a cohort of 259 patients undergoing bronchoscopy at the Medical University of Graz, Austria, between May 2023 and November 2023. The predefined number of cases (= true GM positives) and controls (= true GM negatives) was 50 and 200, respectively. Thus, the first 50 cases and 200 controls that occurred within the observational period were included in the study.

IPA classifications was performed according to the 2020 EORTC/MSG consensus definitions, as well as the recently published 2024 FUNDICU consensus criteria [20, 28]. Cases fulfilling criteria for probable IPA according to EORTC/MSG and/or FUNDICU were considered probable cases for statistical purposes. All samples included in the study were tested for presence of fungi and fungal antigens as part of the routine work-up before being stored at − 20 °C until subsequent testing by the clarus AGM prototype. For extended storage (> four weeks), samples were kept at − 80 °C until further testing. Collected samples were tested using the clarus AGM prototype, within four weeks of the last Platelia AGM, following the manufacturer's recommendations [29]. In case clarus AGM prototype testing was performed more than four weeks after the routine GM testing by the Platelia AGM assay, the Platelia AGM test was repeated to ensure a four-week window between the two tests.

For the clarus AGM prototype testing procedure, 100 µl of pre-treatment buffer (4% EDTA solution; 0.2% ProClin) was mixed with 300 µl of each sample and heated in a dry heat block (Grant QBD2, Grant Instruments, Amsterdam, NL) at 120 °C for 7 min (acceptable range 6–8 min). After centrifugation at 14,000×*g* (acceptable range from 10,000 to 14,000×*g*) for 5 min, 100 µl of each sample was transferred to a microtiter plate, sealed, and incubated at 37 °C for 60 min. The plate was then washed five times with 300 µl of 1 × wash buffer (20 × Enzyme Immunoassay wash buffer; contains 0.4% Tween20, 0.2% ProClin) using an automated miroplate washer (HydroFlex microplate washer, TECAN Grödig, Austria), tapping to remove excess buffer. Conjugate (100 µl) was added to each well, sealed, and incubated at 37 °C for 60 min, followed by another round of washing. Substrate (Tetramethylbenzidine, 100 µl) was added, and after a 30-min incubation at 37 °C, stop solution (< 5% methanesulfonic acid, 100 µl) was added to each well. The plate was gently shaken to ensure homogeneous solution distribution, and absorbance was measured at 450 nm and 620/630 nm using a two-wavelength microplate reader (Sunrise microplate reader with Magelan 7.2 software, TECAN Grödig, Austria) within 15 min after adding stop solution. Corrected optical density values were calculated, and Enzyme Immunoassay units were determined.

The ethical committee of the Medical University of Graz (35–204 ex 22/23) approved the study protocol and all study related procedures. All procedures were performed according to the Declaration of Helsinki. Statistical analyses were performed using IBM SPSS Statistics Version 29 (SPSS Inc., Chicago, IL, USA). Sensitivity and specificity for IPA, defined by criteria for probable versus no IPA, were determined using 2 × 2 tables. Calculations were based on manufacturer-recommended positivity cutoffs: ≥ 0.2 optical density index (ODI) for clarus AGM prototype and ≥ 1 ODI for BALF for Platelia AGM. Additionally, an assessment for Platelia AGM was conducted using a cutoff of ≥ 0.5 ODI. Receiver operating characteristic (ROC) curve analyses were performed for Platelia AGM and clarus AGM prototype with area under the curve (AUC) values and 95% confidence intervals (CI) calculated for the outcomes of probable IPA versus no IPA in the study cohort. To minimize confounding factors, additional analysis (sensitivity, specificity, AUCs for both assays) was carried out by reclassifying probable IPA vs. no IPA after excluding AGM as a mycological criterion. Optimal cutoff values for clarus AGM prototype were determined using Youden’s index. Fisher's Exact and Chi-square tests were employed as appropriate to compare sensitivities and specificities. The significance of the AUC was compared using the Hanley & McNeil method. A two-sided p-value of 0.05 was considered as the threshold for statistical significance.

## Results

### Baseline Characteristics

Out of 259 samples obtained, 20% (53/259) were classified as probable IPA according to 2020 EORTC/MSG criteria and 2024 FUNDICU criteria, while the remaining 80% (206/259) were classified as having no IPA. No patient fulfilled the criteria for proven IPA. Demographic breakdown of the patients included 149/259 females (58%) and 110/259 males (42%), with a median age of 65 years, ranging from 20 to 89 years (Table 1). Nine percent (22/259) had hematological diseases, 1% (3/259) were SOT recipients, 30% (77/259) required ICU admission, 29% (75/259) had solid organ malignancies and 31% (82/259) presented with other underlying conditions. Among all samples, 9% (22/259) were collected from patients with ongoing mold-active antifungal prophylaxis at time of BALF sampling.

**Table 1:** Study cohort characteristics for BALF samples

Baseline characteristics for BALF samples	Probable IPA (n = 53)	No IPA (n = 206)	Total (n = 259)
*Sex*, n (%)			
Female	20 (38%)	90 (44%)	149 (58%)
Male	33 (62%)	116 (56%)	110 (42%)
*Median age in years (range)*	66 (23–82)	65 (20–89)	65 (20–89)
*Underlying diseases/conditions*, n (%)			
Hematological malignancy	3 (6%)	19 (9%)	22 (9%)
Solid organ transplantation	2 (4%)	1 (1%)	3 (1%)
Need for intensive care treatment	24 (45%)	53 (26%)	77 (30%)
Solid organ malignancy	13 (25%)	62 (30%)	75 (29%)
Other	11 (21%)	71 (35%)	82 (31%)
*Antifungal prophylaxis at time of sampling*, n (%)	13 (25%)	9 (4%)	22 (9%)
*Mycological test results from BALF*, n (%)			
*Aspergillu*s growth in BALF culture	14 (26%)	13 (6%)	27 (10%)
*Aspergillus* spp. BALF PCR^§^	2/6 (33%)	1/47 (2%)	3/53 (6%)
AGM ≥ 0.5 ODI Platelia	49 (92%)	51 (25%)	100 (39%)
AGM ≥ 1.0 ODI Platelia	45 (85%)	19 (9%)	64 (25%)
AGM ≥ 0.20 ODI clarus prototype	51 (96%)	53 (26%)	104 (40%)

*AGM Aspergillus* Galactomannan, *BALF* bronchoalveolar lavage fluid, *IPA* invasive pulmonary aspergillosis, *PCR* polymerase chain reaction, *ODI* optical density index

^§^AsperGenius (PathoNostics, Maastricht, The Netherlands)

### Analytic Performances

**Fig. 1:**
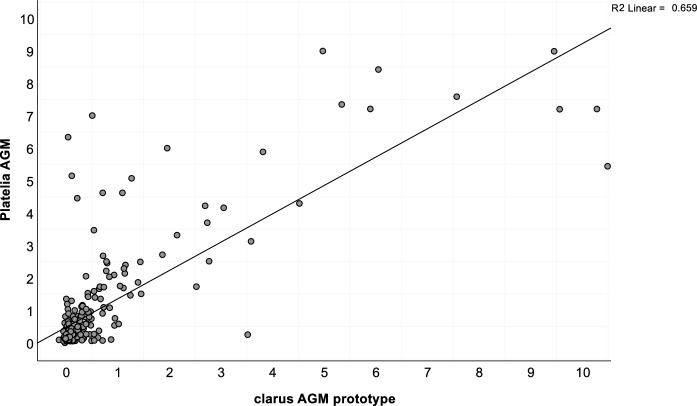
Scatterplot highlighting the correlation of Platelia AGM assay and clarus AGM prototype assay from BALF (n = 259). ODI = optical density index

Spearman's correlation analysis demonstrated a strong positive correlation between the clarus AGM prototype and Platelia AGM results (rho = 0. 727, *p* < 0.001) (Fig. 1) in the overall study cohort.

When distinguishing between probable IPA and no IPA, the clarus AGM prototype performance (Table 2) with the manufacturer-recommended cutoff of ≥ 0.20 ODI achieved a sensitivity of 96% (51/53) and a specificity of 74% (153/206). Optimizing diagnostic discriminatory power using Youden’s index resulted in a cutoff value of ≥ 0.26 ODI for clarus AGM prototype. With this adjustment, sensitivity remained 96% (51/53), while specificity increased to 81% (167/206). The specificity of clarus AGM prototype with ≥ 0.26 ODI was significantly lower compared to Platelia AGM with ≥ 1.0 ODI (*p* = 0.003), while the difference in sensitivity was not statistically significant (*p* = 0.09).

**Table 2:** Sensitivity and specificity comparison of Platelia AGM and clarus AGM prototype for diagnosing probable IPA in BALF, with and without Platelia AGM test results as a mycological criterion

	Sensitivity (n = 53)	Specificity (n = 206)
*Probable IPA versus no IPA classification*		
AGM ≥ 0.2 ODI clarus prototype	96% (51)	74% (153)
AGM ≥ 0.26 corrected ODI clarus prototype	96% (51)	81% (167)
AGM ≥ 0.5 ODI Platelia	92% (49)	75% (155)
AGM ≥ 1.0 ODI Platelia	85% (45)	91% (188)

	Sensitivity (n = 15)	Specificity (n = 206)
*Probable IPA versus no IPA classification after exclusion of GM as mycological criterion*		
AGM ≥ 0.2 ODI clarus prototype	93% (14)	74% (152)
AGM ≥ 0.26 ODI clarus prototype	93% (14)	81% (167)
AGM ≥ 0.5 ODI Platelia	93% (14)	76% (157)
AGM ≥ 1.0 ODI Platelia	93% (14)	92% (190)

*AGM Aspergillus* Galactomannan, *BALF* bronchoalveolar lavage fluid, *IPA* invasive pulmonary aspergillosis, *ODI* optical density index

For the Platelia AGM comparator test (used for IPA classification), sensitivity was 85% (45/53) and specificity was 91% (188/206) when utilizing a cutoff of ≥ 1.0 ODI, while sensitivity was 92% (49/53) and specificity was 75% (155/206) when using the ≥ 0.5 ODI cutoff.

When combining clarus AGM prototype ≥ 0.26 with Platelia AGM ≥ 1.0, test performance sensitivity remained unchanged with 96% (51/53) with either/or both tests resulting positive, while specificity increased to 94% (194/206) when positive results of both tests were required.

**Fig. 2:**
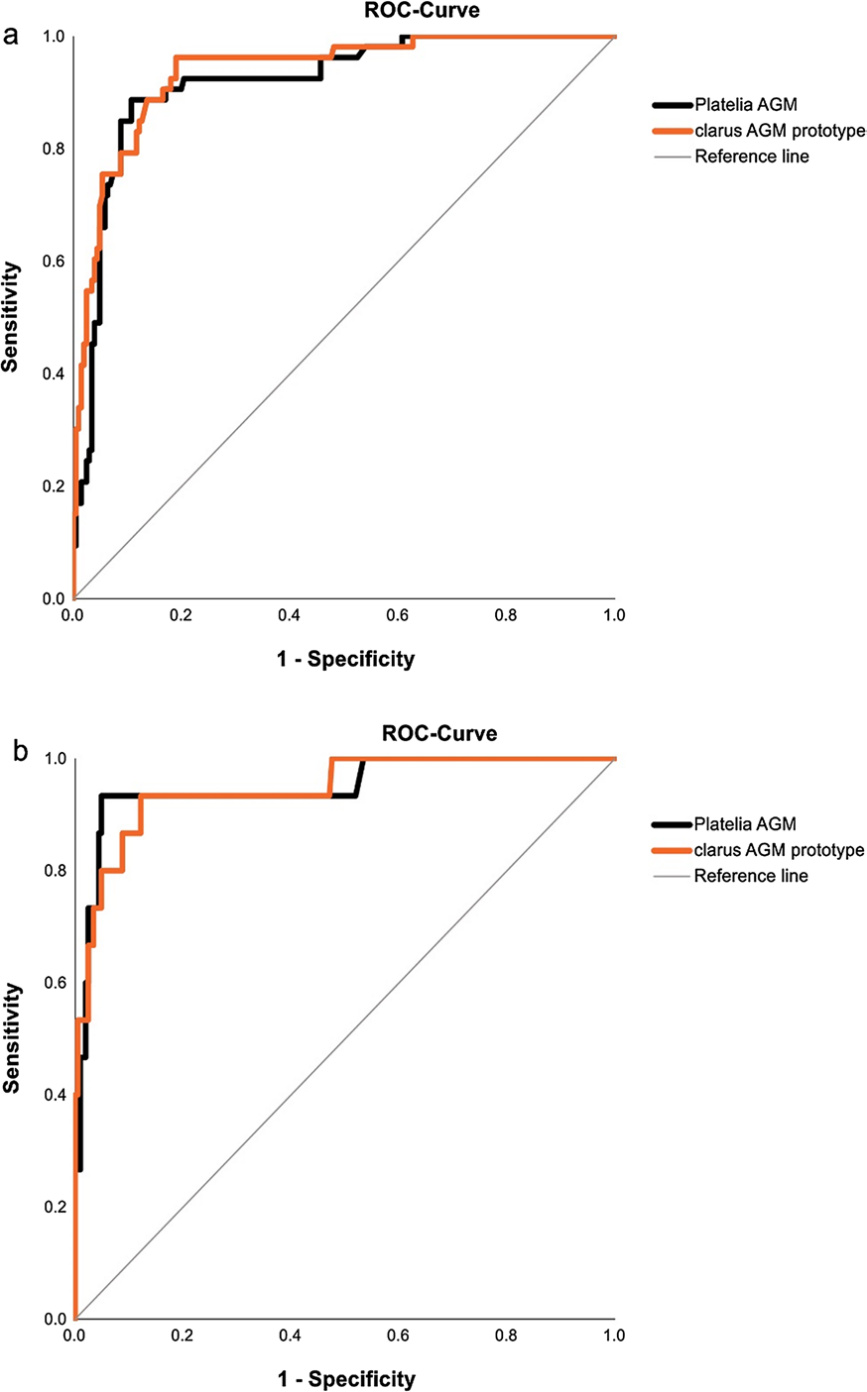
Receiver Operating Characteristic (ROC) curves of Platelia AGM assay and clarus AGM prototype assay from BALF for (**a**) all included samples of probable IPA versus no IPA (n = 259) and (**b**) only samples of cases, diagnosis (IPA vs. no IPA) of which was independent of Platelia AGM results (n = 221)

ROC curve analysis demonstrated an AUC of 0.936 (95% CI of 0.901–0.971) for clarus AGM prototype and 0.918 (95% CI of 0.876–0.959) for the Platelia AGM assay (Fig. 2a).

Nine percent (22/259) of the samples were obtained from patients undergoing mold-active antifungal prophylaxis. Among these, 13 had probable IPA. Using for clarus AGM prototype a cutoff value of ≥ 0.2 ODI, the sensitivity was 92% (12/13), with a specificity of 56% (5/9). Increasing the cutoff value to ≥ 0.26 ODI maintained the sensitivity at 92%, while the specificity increased to 67% (6/9). The AUC for clarus AGM prototype was numerically but not significantly lower with antifungal prophylaxis (0.863 (95% CI 0.711–1) compared to patients without prophylaxis 0.951 (95% CI 0.920–0.981; *p* = 0.27). The AUC for the Platelia AGM in patients with ongoing mold-active antifungals was 0.940 (95%CI 0.835–1), and not significantly different from the performance of the clarus AGM prototype in this cohort (*p* = 0.42).

When classifying probable IPA vs. no IPA based solely on *Aspergillus* culture and PCR (Table 2), only 19/53 probable IPA cases remained probable IPA cases. The AUCs for Platelia AGM and clarus AGM prototype were 0.948 (95% CI 0.882–1) and 0.945 (95% CI 0.884–1), respectively (Fig. 2b).

The Platelia AGM had a sensitivity of 93% (14/15) and a specificity of 92% (190/206) at a cutoff value of ODI ≥ 1.0. Lowering the cutoff to ≥ 0.5 ODI maintained sensitivity at 93% (14/15), with specificity decreasing to 76% (157/206). At the manufacturer-recommended cutoff of ODI ≥ 0.2, the clarus AGM prototype had a sensitivity of 93% (14/15) and specificity of 74% (152/206). Using the calculated optimal cutoff of ODI ≥ 0.26, sensitivity remained 93% (14/15), while specificity increased to 81% (167/206).

## Discussion

In this study, we compared the clarus AGM prototype assay with the validated Platelia AGM assay in BALF samples from a prospectively enriched patient cohort. Our findings revealed a strong correlation between the two assays, with clarus AGM prototype demonstrating comparable sensitivity and specificity to Platelia AGM. Notably, clarus AGM prototype showed promising diagnostic accuracy, suggesting its potential as an accessible and affordable alternative for early detection of IPA.

The clarus AGM prototype assay utilizes a monoclonal antibody targeting a proprietary mix of two different antibodies: the ME-A5 human immunoglobulin G monoclonal antibody (mouse derived), and an undisclosed proprietary antibody with an unreleased GM-Ag target [30]. On the other hand, the Platelia AGM assay employs the rat monoclonal immunoglobulin M antibody EB-A2, specifically directed against *Aspergillus* GM-Ag [31]. Costs are a major factor limiting the potential to implement and to utilize the assay, and therefore timely access to AGM test results, particularly in low- and middle-income settings [21–23, 32]. By providing a reliable alternative to the Platelia AGM assay, the clarus AGM prototype could enhance the diagnostic capabilities across diverse healthcare settings, potentially improving patient outcomes through timely identification and targeted treatment of IA. Early diagnosis is particularly important in the light of several new antifungal compounds that are currently in the pipeline for the successful treatment of aspergillosis [33].

Different antigens may dominate in different stages of IPA, as indicated in previous studies [34], which implies that tests targeting different antigens may offer advantages and disadvantages at various disease stages. This variability explains why, despite an overall strong correlation, the results between Platelia AGM and clarus AGM prototype assays differed in some cases, as they may recognize similar but not identical antigens. The challenge of immunological assays lies in the variability of antibodies, underscoring the importance of considering the antibody's species of origin, which can significantly impact test specificity and sensitivity. Utilizing a combination of tests might be beneficial in capturing the complexities of IPA progression, as shown in our cohort where sensitivity and specificity could be increased by using different combination scenarios.

We observed a strong correlation between the clarus AGM prototype and the Platelia AGM test results, indicating that the clarus AGM prototype exhibits similar reliability as the Platelia AGM for the detection of GM. This observation is particularly noteworthy as the diagnosis of IPA was initially established using the Platelia AGM assay. The clarus AGM prototype yielded a trend towards a higher sensitivity (96% vs. 85%) while specificity was markedly lower (74% vs 91%) for differentiating between probable IPA vs no IPA versus Platelia AGM with cutoff of ≥ 1.0 ODI. Using a cutoff of ODI ≥ 0.26 identified by Youden´s index, specificity of the clarus AGM prototype increased to 81% with preserved sensitivity. These findings are comparable with data of a systematic review evaluating the value of the Platelia BALF-AGM in the diagnosis of IPA in hematological patients, which reported a pooled sensitivity of 82% (70–91%) and a specificity of 92% (85–96%) [35]. They are also comparable with performances reported in multicenter studies for the BALF *Aspergillus* GM LFA point of care test in intensive care unit patients at risk for IPA [26, 27, 36]. In contrast to BALF testing, serum testing for AGM may have more limited application in non-neutropenic patients, where observed sensitivities are generally low [27, 37]. This was also confirmed for the clarus AGM prototype in another single-center, cross-sectional study, where the clarus AGM prototype display lower sensitivity and specificity compared to Platelia AGM with the manufacturer recommended cutoff [38]. Upon adjusting the cut-off values, clarus AGM prototype demonstrated enhanced diagnostic accuracy, suggesting the necessity for additional exploration into its clinical performance when testing serum samples.

To further investigate the diagnostic performance of both the Platelia AGM and the clarus AGM prototype assays, we performed a subanalysis excluding GM-Ag results as a mycological criterion for clinical classification. In this subanalysis, where *Aspergillus* culture and PCR were exclusively used as mycological evidence for the classification of IPA, the diagnostic performance of both assays in discriminating between probable IPA and non-IPA cases remained robust. Notably, in this subanalysis, both the clarus AGM prototype and Platelia AGM showed a sensitivity of 93%, with clarus exhibiting 81% specificity at ≥ 0.26 ODI cutoff and Platelia showing 92% specificity at ≥ 1.0 ODI cutoff.

Despite these insights, our study has several limitations. First, caution is warranted when interpreting clinical performance data and AUC values due to the study design involving a cohort’s enrichment with IPA cases, many of which also tested positive for Platelia AGM test results. This correlation could potentially overestimate the performance of Platelia AGM.

Future research involving multiple centers could provide a more comprehensive evaluation of these assays across diverse patient populations, thereby enhancing the confidence and applicability of our findings. Additionally, the limited number of culture-positive cases in our study underscores the need for further investigation into the potential of the clarus AGM prototype assay to distinguish between different causative Aspergillus species. In conclusion, our study indicates that the clarus AGM prototype could serve as a viable alternative in GM-Ag testing of BALF samples of patients at risk of IPA. As our study suggests a cutoff of an ODI ≥ 0.26 potentially leading to an increased specificity by preserved sensitivity, further studies are warranted to fully explore its diagnostic potential. Globally, there remains a critical need for accessible and reliable antigen-based assays to enhance our diagnostic capabilities for IPA.
